# Asking New Questions with Old Data: The Centralized Open-Access Rehabilitation Database for Stroke

**DOI:** 10.3389/fneur.2016.00153

**Published:** 2016-09-20

**Authors:** Keith R. Lohse, Sydney Y. Schaefer, Adam C. Raikes, Lara A. Boyd, Catherine E. Lang

**Affiliations:** ^1^School of Kinesiology, Auburn University, Auburn, AL, USA; ^2^School of Biological and Health Systems Engineering, Arizona State University, Tempe, AZ, USA; ^3^Department of Health, Physical Education and Recreation, Utah State University, Logan, UT, USA; ^4^Department of Physical Therapy, University of British Columbia, Vancouver, BC, Canada; ^5^Program in Physical Therapy, Washington University School of Medicine in St. Louis, St. Louis, MO, USA; ^6^Program in Occupational Therapy, Washington University School of Medicine in St. Louis, St. Louis, MO, USA; ^7^Department of Neurology, Washington University School of Medicine in St. Louis, St. Louis, MO, USA

**Keywords:** stroke, rehabilitation, informatics

## Abstract

**Background:**

This paper introduces a tool for streamlining data integration in rehabilitation science, the *C*entralized *O*pen-*A*ccess *R*ehabilitation database for *S*troke (SCOAR), which allows researchers to quickly visualize relationships among variables, efficiently share data, generate hypotheses, and enhance clinical trial design.

**Methods:**

Bibliographic databases were searched according to inclusion criteria leaving 2,892 titles that were further screened to 514 manuscripts to be screened by full text, leaving 215 randomized controlled trials (RCTs) in the database (489 independent groups representing 12,847 patients). Demographic, methodological, and statistical data were extracted by independent coders and entered into SCOAR.

**Results:**

Trial data came from 114 locations in 27 different countries and represented patients with a wide range of ages, 62 year [41; 85] [shown as median (range)] and at various stages of recovery following their stroke, 141 days [1; 3372]. There was considerable variation in the dose of therapy that patients received, 20 h [0; 221], over interventions of different durations, 28 days [10; 365]. There was also a lack of common data elements (CDEs) across trials, but this lack of CDEs was most pronounced for baseline assessments of patient impairment and severity of stroke.

**Conclusion:**

Data integration across hundreds of RCTs allows clinicians and researchers to quickly visualize data from the history of the field and lays the foundation for making SCOAR a living database to which researchers can upload new data as trial results are published. SCOAR is a useful tool for clinicians and researchers that will facilitate data visualization, data sharing, the finding of relevant past studies, and the design of clinical trials by enabling more accurate and comprehensive power analyses. Furthermore, these data speak to the need for CDEs specific to stroke rehabilitation in randomized controlled trials.

**PROSPERO 2014:**

CRD42014009010

## Introduction

The information architecture in rehabilitation science is poor (1). For example, randomized controlled trials (RCTs) are the basic “unit” of information that guide clinical practice. Yet when clinicians and scientists want to ask a very basic question of these data, they are published: (1) across a wide spectrum of journals and formats that often have limited access (e.g., payment required for access); (2) embedded potentially in text, tables, figures, or even supplemental materials; and (3) with very few common data elements (CDEs) reported across studies (2, 3). Thus, despite the tremendous time and financial burdens associated with even a single RCT, the resultant data lack a consistent structure. This lack of structure is an unnecessary barrier to integration in future scientific and clinical practice. Efforts to streamline data integration should increase the transparency and visibility of comprehensive bodies of evidence, rather than a single study, to better inform clinically relevant questions such as, “How do therapy outcomes change with increased time in therapy?” or “How variable are outcomes, historically, for specific parameters of therapy?”

We now introduce one such tool for streamlining data integration: the *C*entralized *O*pen-*A*ccess *R*ehabilitation database for *S*troke (SCOAR). In short, SCOAR is a central repository for summary statistics from RCTs. SCOAR currently contains data from a systematic review and extraction of papers from 1981 to early 2014 (described in detail below), but the goal of SCOAR is much bigger: to create a “living” database where new data can be added as clinical trials are completed. Imposing such an architecture (4) on clinical trial data would allow basic and clinical scientists to (1) quickly and easily visualize relationships among variables, (2) efficiently share data, (3) generate hypotheses based on noticeable patterns or even “gaps” in the current data, (4) search the current literature from the data up (rather than key-terms down), and (5) improve clinical trial design through more accurate and comprehensive power analyses.

Generally speaking, the goal of SCOAR is to improve the design of future clinical trials by giving researchers fast and easy access to the historical range of effect-sizes, based on thousands of stroke patients who received therapies of different types, different doses, at different times, and were measured on different outcomes. From our perspective, the effort associated with the design, implementation, and dissemination of randomized clinical trials *deserves* an information architecture that supports and increases their visibility. In the current paper, we (1) explain the systematic search and data extraction that led to the creation of SCOAR; (2) present summary statistics for the major variables in SCOAR, including the geographical reach, to understand how SCOAR data represent research in stroke rehabilitation; and (3) based on the lack of CDEs we find across many variables, we argue for a consistent set of CDEs in rehabilitation trials (CDEs to describe participants, methodology, and outcomes). SCOAR lays the foundation for an information architecture that captures some of the complex and multivariate nature of neurorehabilitation. Most importantly, this information architecture is scalable, making it easy to add new data as new trials are published.

## Materials and Methods

### Systematic Review

**Figure 1:**
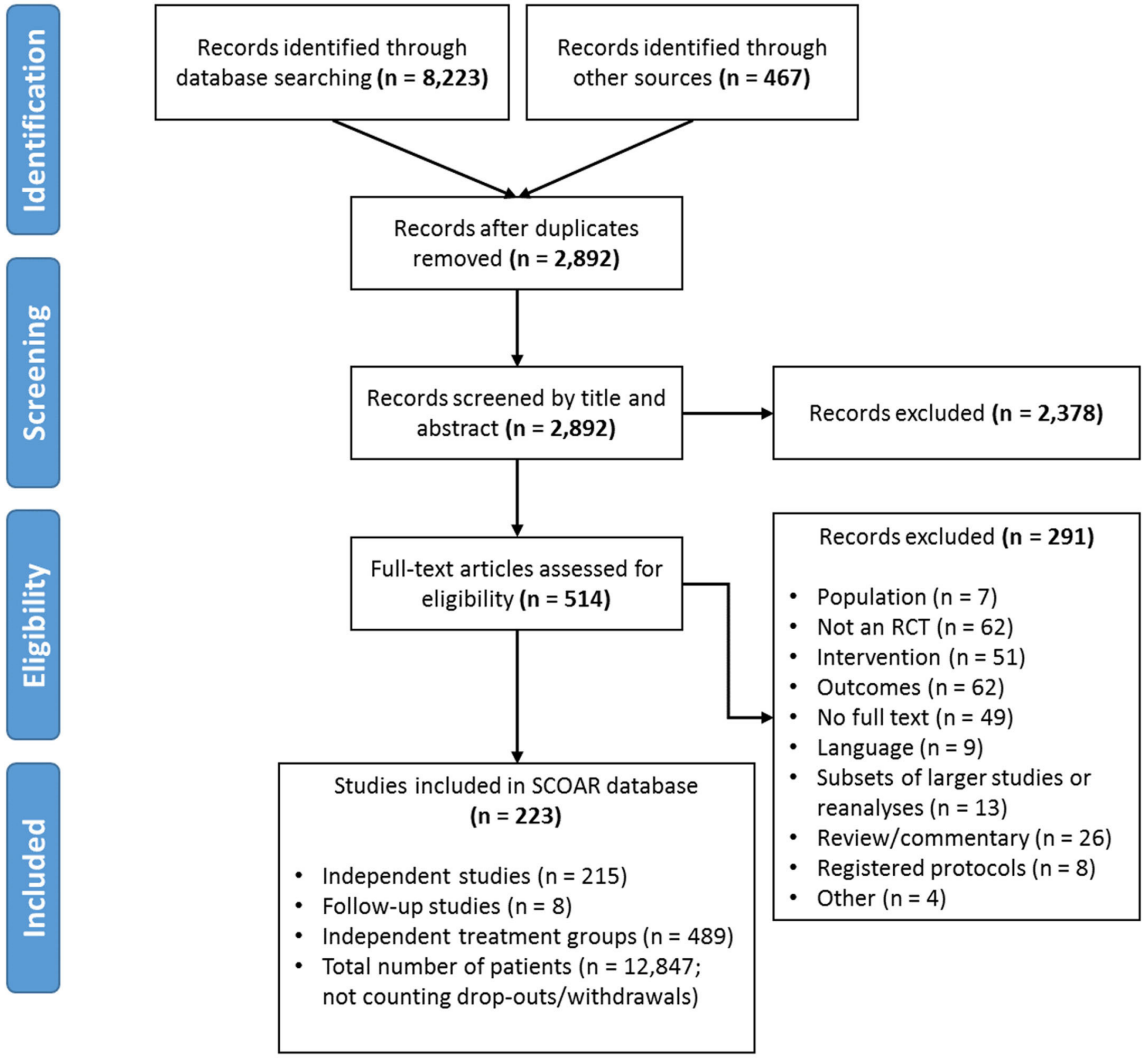
**PRISMA Flow diagram showing the manuscript screening process**. At the eligibility assessment, manuscripts were excluded if the population was not stroke, there was no random assignment to at least two different groups, if the intervention did not meet our population, intervention, control, and outcome criteria, if the outcomes were not a clinical measure of function/impairment, if no full text of manuscript was available (e.g., restricted access or the text only existed in abstract form), if the data in the manuscript came from a larger study/reanalysis of a study that was already in the database, if the manuscript was a review, commentary, or a trial protocol (rather than a trial itself), or if it was not clear how the study related to an existing study in the database (i.e., possibly the same patients being analyzed twice; coded as “other”).

A systematic search was undertaken in May 2014 (full details in PROSPERO 2014:CRD42014009010) using the following databases: MEDLINE, EMBASE, Cochrane CENTRAL, Cochrane-CDSR, and CINAHL. Outside references were also incorporated from previous reviews (5, 6) (Figure 1). After removing duplicates, 2,892 remaining titles and abstracts were screened by independent coders (two pairs of trained graduate students) based on the following inclusion/exclusion criteria (7):

#### Population

Human adults with stroke, >18 years of age, with a motor impairment as a result of stroke. Any etiology was included (e.g., ischemic, hemorrhagic, sub-arachnoid hemorrhage) provided that the study identified patients as having a stroke or cerebrovascular vascular accident. Groups with mixed neurological impairments (e.g., patients had either stroke or TBI) were excluded. (In the RCTs we found, the majority of groups were characterized as having first-ever unilateral strokes, without other neurological conditions, but these were not criteria for inclusion.)

#### Intervention

Any physical or occupational therapy interventions that required active movement on the part of the participant were included. RCTs that used stimulation techniques were allowed (e.g., FES, TMS, tDCS) if combined with active movement. Studies with strictly pharmaceutical interventions (e.g., to treat spasticity) were excluded.

#### Control

All studies had to be RCTs and studies were required to explicitly state random assignment to groups. The condition nominally identified as “control” by the authors was coded as control, or if a group received “conventional care,” “routine therapy,” or “standard care” without being specifically named as control, it was assumed that this was the control condition. All other conditions were labeled as “experimental.”

#### Outcome

Only empirically validated assessments of impairment or functional motor capacity (i.e., activity limitations) administered by the clinician were taken as outcomes (e.g., no self-report measures, no neuro-imaging measures, no study-specific kinematic/kinetic measures). Note that studies could include these other types of measures, but only measures of impairment or function were extracted for inclusion in the database at this time.

If there was disagreement between the coders, the first author (Keith R. Lohse) provided a tie-breaking vote on inclusion or led discussion until agreement was reached. The same PICO inclusion/exclusion criteria were also applied to the full text review. During the full text review, the authors systematically extracted data from the manuscripts (described below in Section “Data Extraction”). During the extraction, concerns emerged regarding how to consider statistically related data within SCOAR. In other words, SCOAR is designed to longitudinally track groups over time (viz., from baseline assessment, to terminal assessment, to the last long-term follow-up) and, therefore, studies with long-term follow-up data, subsets of data, or re-analyzed data published in separate manuscripts presented a unique challenge. To maintain independence of the data for statistical analysis, any given group of patients should only appear in the SCOAR database once. Thus, subsets and re-analyses were excluded (*n* = 8) or if the relationship between manuscripts was unclear (*n* = 5), those studies were excluded as well. However, if a paper published a long-term follow-up or reported usable outcome measures in a separate manuscript (*n* = 8), then these data were grouped together in the SCOAR database [e.g., Ref. (8–11)], such that independence between groups is maintained. As shown in Figure 1, there were a number of studies for which the full text could not be obtained (*n* = 49), or even if the full text was available, there was no available English translation (*n* = 9). (At present, no effort has been made to translate these non-English texts, but with adequate translation, the data from these studies may be eligible for inclusion following review.) In total, SCOAR has summary statistics from 12,847 patients in 489 independent groups (as of 2016-03-31).

### Data Extraction

Separate from the pairs of graduate students who screened by title and abstract, three authors (Keith R. Lohse, Sydney Y. Schaefer, Adam C. Raikes) extracted data by hand from electronic copies of manuscripts using a standardized data extraction form. These extracted values were then entered by hand into a spreadsheet. Discrepancies between extractors were discussed until consensus was reached. A copy of the data extraction form is listed in Data Sheet S1 in Supplementary Material and an explanation of all current SCOAR variables is included in the data dictionary, Data Sheet S2 in Supplementary Material.

One author (Keith R. Lohse) extracted outcome data for either the primary outcome or the first usable outcome, when no primary outcome was stated or the primary outcome was not usable. As per the PICO criteria, a usable outcome measure was defined as an assessment given by a therapist that was a clinical measure of motor impairment and/or function. Two authors (Sydney Y. Schaefer, Adam C. Raikes) also reviewed all of the articles to extract all additional data related to the Fugl–Meyer Assessment (FMA, upper-extremity portion, a measure of upper limb impairment) and gait speed (including the 10-m walk test, or variations thereof, a measure of walking function). This second set of outcomes was extracted regardless of whether the FMA or gait speed measure was stated as a primary outcome. FMA and gait speed measures were chosen for this second extraction because they were the most commonly reported measures for those two domains in previous work (12).

In extracting these data, the relevant outcome data were often clearly presented. For instance, baseline means and SDs were consistently reported in the text (i.e., the pre-intervention assessment, reported in 93 and 83% of cases), whereas terminal means (i.e., the most immediate post-intervention assessment; calculable for 93% of cases) sometimes had to be calculated by the data extractors from reported change scores (12% of calculable cases). Additionally, terminal SDs (calculable for 72% of cases) were sometimes estimated by the data extractors from figures or reported confidence intervals (3% of calculable cases).

Other data were often not reported in the text and, therefore, had to be estimated by the data extractors. For instance, the time scheduled for therapy was typically estimated from written descriptions (e.g., “1 h per day, 5 days per week for 4 weeks” = 20 h of scheduled therapy). An estimate of the time scheduled for therapy was calculable in 74% of cases. Although some studies reported repetitions of movements (13, 14) or active time in therapy (15, 16), the most common metric was the time scheduled for therapy. Although time scheduled for therapy is likely to be a poor indicator of the actual amount of therapy received (17), it is positively correlated with the amount of therapy received and was the most common metric reported across trials. Constraint-induced movement therapies (18, 19) or other “forced-use” therapies (20) were problematic for estimating the time scheduled for therapy because they often do not specify the amount of time actually spent in constraint or forced-use. In line with previous work (6), we calculated time under constraint in three different ways: (1) counting 100% of constraint time as time in therapy, (2) counting 50% of constraint time as time in therapy, and (3) counting 0% of constraint time as time in therapy. The 50% time calculation is preferred because it has the most plausible assumptions (i.e., some, but not all, of constraint time is spent using the affected extremity), but all three calculations are available to researchers in the database. We further note that some constraint studies reported constraint for a “percentage of waking hours,” and these percentages were converted to hours based on 16 waking hours per day.

### Estimation of Within-Group Effect Size

Compared to effect-size calculations in other rehabilitation meta-analyses (5, 6), the default effect-size in SCOAR represents the change within groups over time rather than a difference between groups at a specific time point. Calculating a standardized effect-size for within-group change is important, because these effect-sizes allow for the greatest flexibility in integrating changes across studies using the most data [i.e., each group’s improvement (or decrement) is normalized to their baseline at the beginning of the intervention]. Calculation of these within-group changes creates some unique challenges for meta-analysis (see below), but all effect-size calculations were for a Cohen’s *d* as described in Borenstein et al. (21).

(1) $$d=\frac{{\bar{y}}_{1}-{\bar{y}}_{2}}{s_{\mathrm{pooled}}}$$

where *s*_pooled_ is the between-person SD pooled between the two different time points to create a single estimate of the between-person variance.

(2) $$s_{\mathrm{pooled}}=\sqrt{\frac{\left(n_{1}-1\right)s_{1}^{2}+\left(n_{2}-1\right)s_{2}^{2}}{\left(n_{1}+n_{2}-2\right)}}$$

Thus, $s_{1}^{2}\text{ and }s_{2}^{2}$ refer to the variance at the baseline and the terminal assessment, respectively. In 28% of the total cases, the variance was not estimable at the terminal assessment. However, in 16% of the total cases the baseline SD was available and used in the calculation of *d* when the terminal SD was not available. In fewer cases (1% of total cases), the variance at baseline was zero (e.g., all participants had a Functional Ambulation Category of zero due to a floor effect in the outcome measure). In those cases, the terminal SD was used in the calculation of *d*.

Finally, given the wide range of the effect-sizes and sample sizes we observed in the data, we transformed these effect-sizes from Cohen’s *d* to Hedges’ *g*. Cohen’s *d* is biased to overestimate the underlying effect-size in small samples whereas Hedges’ *g* is a more conservative and unbiased calculation of the effect-size in which the *d* value is reduced proportional to the sample size (21). Subtraction in the effect-size calculations was arranged such that positive values in SCOAR always reflect improvement relative to baseline.

Although the default effect-sizes in SCOAR represent the normalized improvement within a group over time, we should point out that SCOAR also contains the sample size, mean, and SD for all groups at the baseline, terminal, and follow-up assessment (if applicable). Having these descriptive statistics for each group at each time point allows researchers to readily calculate between-group effect sizes if those effects are more relevant to their research question. Thus, by extracting the sample size, mean, and SD at each time point, SCOAR allows researchers to calculate outcomes in three different ways: (1) a standardized effect-size showing change within a group over time (the default SCOAR effect-size), (2) a standardized effect-size showing the difference between groups at a single point in time, or (3) the original “raw” units of the outcome measure. Although using original units precludes combining different outcomes into a single analysis, this is a sensible option when restricting outcomes to a single type (i.e., measures of gait speed can all be expressed in terms of meters per second, so there is no need to normalize).

### Estimation of the Correlation between Time Points and Effect-Size Variance

In order to conduct quantitative meta-analyses with these data, we also need to calculate the variance of the individual effect-sizes. For statistically dependent “within-subject” data, the correlation between time points, *r*, is required for an accurate estimation of effect-size variance, *V_d_* (21).

(3) $$V_{d}=\left(\frac{1}{n}+\frac{d^{2}}{2n}\right)2\left(1-r\right)$$

The correlation between baseline and terminal (or follow-up) scores was never reported in any of the included RCTs. We were, however, able to estimate the correlation between baseline and terminal assessments from studies that provided either (A) individual patient data or (B) SDs of the baseline, terminal, and baseline-to-terminal change scores (21). In this subset of studies (13, 22–34), we observed that the median correlation was *r* = 0.87, IQR = (0.70, 0.93) and the minimum correlation was *r* = 0.28 between baseline and terminal assessments. As such, we calculated effect-size variance based on the conservative assumption that *r* = 0.5 for all studies (which generally creates larger estimates of variance in the data, widening our confidence intervals). This estimated correlation is, however, an easy value for researchers to manipulate within SCOAR and recalculate their own effect-size variances. Thus, the default effect-size variance in SCOAR is calculated based on *r* = 0.5, which we consider to be a conservative estimate, but researchers can easily recalculate effect-size variances by scaling this correlation up or down.

## Results

Descriptive statistics across the 489 independent groups of participants currently in the database are listed in Table 1. The full database (as of 2016-03-31), the data dictionary, a full reference list all trials, and the Creative Commons license for SCOAR are available from https://github.com/keithlohse/SCOAR. (Note that SCOAR is licensed under a Creative Commons Attribution-ShareAlike 4.0 International License by Keith R. Lohse.)

**Table 1:** **Demographic statistics for the studies included in SCOAR**.

Variable	Mean (SD)	Median (IQR)	Min; Max	Groups with missing values
Mean patient age (years)	62.6 (6.7)	62.4 (57.3; 67.3)	41.30; 85.20	12
Mean days post stroke (days)	509 (652)	141 (31; 840)	1; 3,372	22
Duration of intervention (days)	45 (39)	28 (28; 42)	10; 365	20
Estimated Time Scheduled for Therapy (h)				
Max time calculation	34.2 (45.3)	20.0 (10.0; 36.1)	0.0; 280.0	129
50% time calculation	29.9 (31.6)	20.0 (10.0; 36.0)	0.0; 220.7	129
Min time calculation	25.5 (24.5)	20.0 (10.0; 32.0)	0.0; 220.7	129
Method for Tracking Dose of Therapy – *N* (% out of 489)				
Hours scheduled	307 (63%)			
Time in therapy	26 (5%)			
Active time	37 (8%)			
Repetitions	20 (4%)			
Groups Reporting ITT Analysis	158 (32%)			
*N* per Group at baseline calculation	26 (26)	18 (11; 31)	4; 165	0
*N* per group at terminal calculation	25 (25)	17 (10; 30)	4; 165	6
*N* per Group at follow-up calculation	29 (29)	19 (2; 34)	4; 165	215
Time from baseline to follow-up (days)	178 (137)	180 (88; 206)	31; 1,098	231
Coded as experimental groups	285 (58%)			
Coded as control groups	204 (42%)			

*N per group refers to the number of participants whose data were used in the calculation of the mean and the SD at each time point, not necessarily the number of participants enrolled in/actively participating in the study at that time point (depending on the analytical method used by the authors). Time in days and N per group are rounded to the nearest integer. ITT = intention to treat*.

As shown in Table 1, the data in SCOAR are representative of a wide range of patients (in terms of day-post stroke and age) and different types of interventions (in terms of sample size, duration of the intervention, and dose of therapy given over the intervention). An important point to note are the different methods used for tracking the dose of therapy patients received. Consistent with our previous work (6), the most common metric for tracking dose was the hours of scheduled therapy. The best measures of dose are the time actively spent doing therapy (19, 35) (which was more commonly reported in gait therapies) or the actual repetitions of therapy exercises (14, 36) (which was more commonly reported in trials using robotic assistive devices).

### Geographical Distribution of Studies

**Figure 2:**
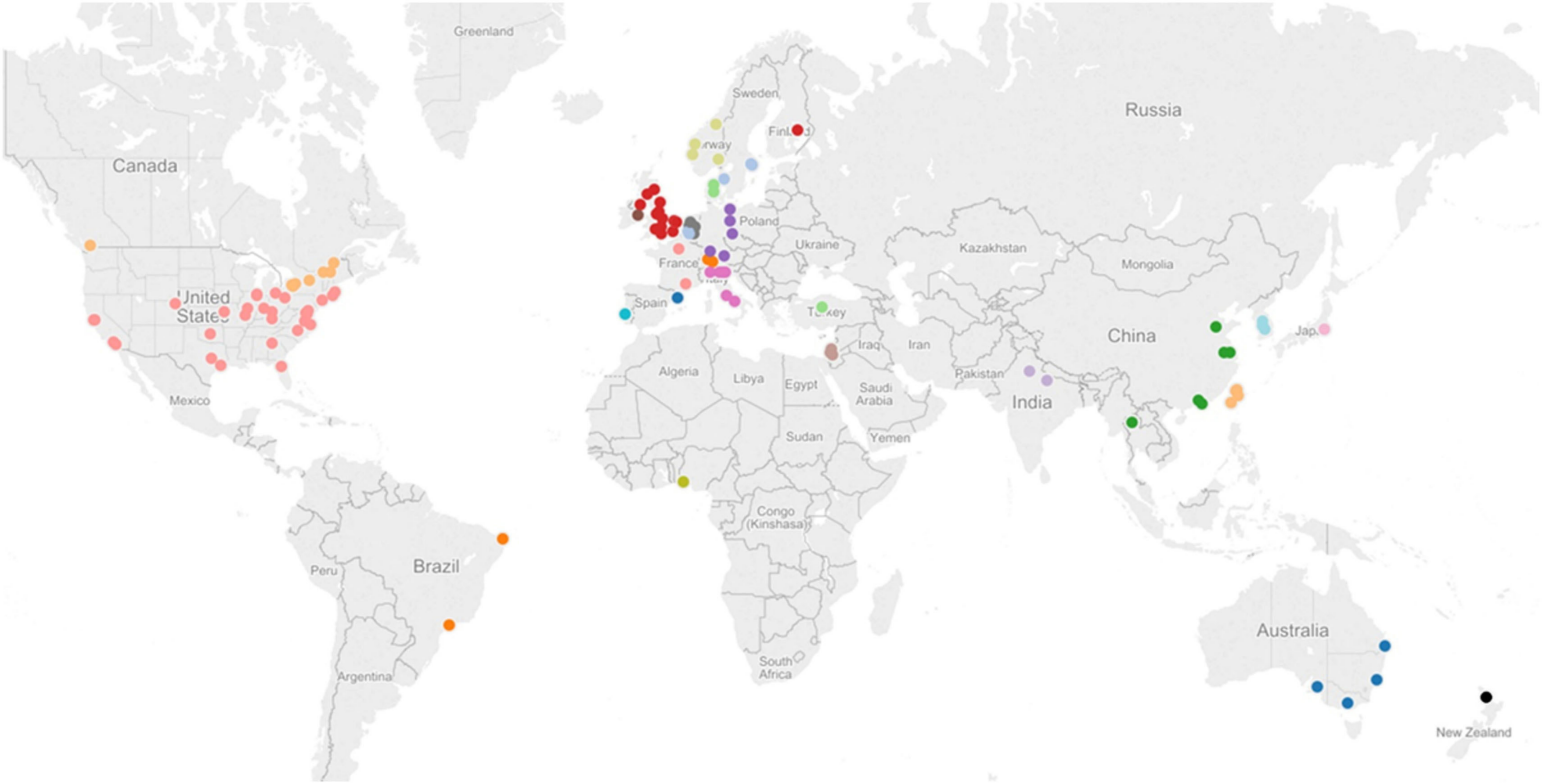
**Geographical distribution of studies in the SCOAR database based on the contact information for the corresponding author**. Each dot represents one city in a given region, but there may be multiple studies from a single city (e.g., there are three different RCTs and six independent groups, from Chicago, IL, USA). An interactive version of this figure is also available from http://tinyurl.com/SCOAR-regions; generated using Tableau 9.0 (Tableau Software; Seattle, WA, USA).

As shown in Figure 2, trials in the SCOAR database come from a wide range of countries. The location of a trial was estimated based on the contact information for the corresponding author. Note that multiple studies may be represented by each dot (if trials were conducted at the same location), so we refer readers to the interactive version of this figure^1^ where they can see the number of trials, number of groups, and the references for each location. Overall, trials came from 114 locations in 27 different countries. From this wide range of trial locations, it does not appear that SCOAR is geographically biased relative to the larger population of stroke rehabilitation trials. The extent to which the population of trials might be biased, however, is an important question. As seen in Figure 2, there is a dearth of trials for regions such as South America, Africa, central Asia, and eastern Europe.

### Common Data Elements for Baseline Assessment and Initial Severity

Across the 489 independent groups of participants, there was a wide range of data elements, but unfortunately there were relatively few CDEs reported across the different studies. This lack of CDEs was especially pronounced for variables measuring the baseline severity of stroke or patient impairment. For example, data describing cognitive status with the Mini-Mental Status Exam (MMSE) were reported for only 152 groups in some form (31% of cases). A mean or median MMSE score was given for 82 (17%) of these groups, whereas 70 (14%) of these groups only reported some cut-off/critical MMSE value in their inclusion/exclusion criteria. The MMSE example was not unusual, however, and was actually the most common CDE for baseline assessment. For a list of the most common baseline assessments and their prevalence (see Table 2).

**Table 2:** **Most common data elements for baseline measures in the 489 independent groups included in SCOAR**.

Measure	Number of groups with mean/median value reported	Number of groups with cut-off stated in I/E criteria only	Not reported
MMSE (or modified) (37)	82 (17%)	70 (14%)	337 (69%)
Ashworth (or modified) (38)	63 (13%)	25 (5%)	401 (82%)
Barthel (or modified) (39)	68 (14%)	2 (<1%)	419 (86%)
FAC (40)	46 (9%)	10 (2%)	433 (89%)
FIM (41)	47 (10%)	2 (<1%)	440 (90%)
Brunnstrom stages (42)	24 (5%)	20 (4%)	445 (91%)
Motricity Index (43)	28 (6%)	7 (1%)	454 (93%)
NIHSS (44)	34 (7%)	0 (0%)	455 (93%)
Berg Balance Scale (45)	34 (7%)	0 (0%)	455 (93%)

*I/E, inclusion/exclusion criteria, MMSE, Mini-Mental Status Exam, FAC, Functional Ambulation Category, FIM, Functional Independence Measure, NIHSS, National Institutes of Health Stroke Scale*.

This lack of CDEs (or at least under-reporting of CDEs) is undoubtedly a major barrier to data integration and meta-analysis, especially for ostensibly common baseline assessments, such as the FIM or NIHSS. We should clarify that it is possible that authors were using these assessments/screening criteria in the actual trial, but they may not have been reporting these measures and we can only extract and analyze the descriptive statistics based on the published information. Indeed, many inclusion/exclusion criteria were vague, with authors often reporting “no excessive spasticity” (implying perhaps the Ashworth scale was used), “no cognitive/communicative impairments” (implying the MMSE may have been used), or “no severe depression” (implying perhaps the Beck Depression Inventory or the Center for Epidemiological Studies depression scale were used). Without more complete reporting, we have no way of knowing which measures were collected and how participants were evaluated. This presents a major gap in stroke rehabilitation knowledge (1).

### Classification and Description of Therapy Types

An unexpected difficulty in the construction of the SCOAR database was in how to categorize/classify different therapeutic interventions. Ideally, researchers could use the SCOAR database to look at effect-sizes as a function of therapy-type or ask other meta-scientific questions about therapy types. Indeed, we were able to extract a short phrase or informative description of the therapy for almost all of the experimental groups (e.g., “CIMT,” “intensive progressive treadmill training”; these descriptions are listed under the “group_desc” variable in the SCOAR database), consistent with TIDieR Criteria (46, 47). For control groups, however, this proved much more difficult, with many descriptions being simply “conventional physiotherapy,” “standard care,” or “routine care.” We are currently pursuing text-mining approaches to better quantify the nature of therapy provided in control-arms of trials. In the included RCTs, however, control groups were under-described compared to experimental groups (48). Looking strictly at the Methods sections of papers, for instance, control groups only had a mean (SD) of 155 (112) words and 0.82 (1.9) references dedicated to their description. Experimental groups, conversely, had 271 (159) words and 1.77 (3.4) references dedicated to their description. Example control group descriptions are:

“All participants received standard rehabilitation, including 40 minutes each of physiotherapy and occupational therapy, given once per day, five days per week for six weeks, by the rehabilitation team. [… *sentence about experimental group omitted*…] The control group had visits and discussions of 20 minutes at least three times per week instead.” (49)

“All participants received the site’s conventional inpatient rehabilitation. They also performed a daily 10-m walk (or shorter distance walk until 10 m was feasible) as part of a physical therapy session.” (50)

“All participants received a duration-matched intervention for 90 to 105 minutes/day, 5 days/week for 4 weeks. The CT group received an intensive therapist-administered control therapy matched in duration with the RT groups. Occupational therapy techniques used in the treatment protocols included neurodevelopmental treatment, muscle strengthening, fine-motor training, and functional task training.” (14)

Adequate descriptions of control therapies are critical, not only because the SCOAR database spans three decades and “routine” therapy has changed considerably over that period, but also because it is impossible to compare the efficacy of a treatment across trials if the control groups to which the treatment is compared are very different. At the moment, the under-describing of control therapies allows for very different interventions to all be categorized as “standard care” in different trials.

### Classification and Common Data Elements in Outcome Measures

**Figure 3:**
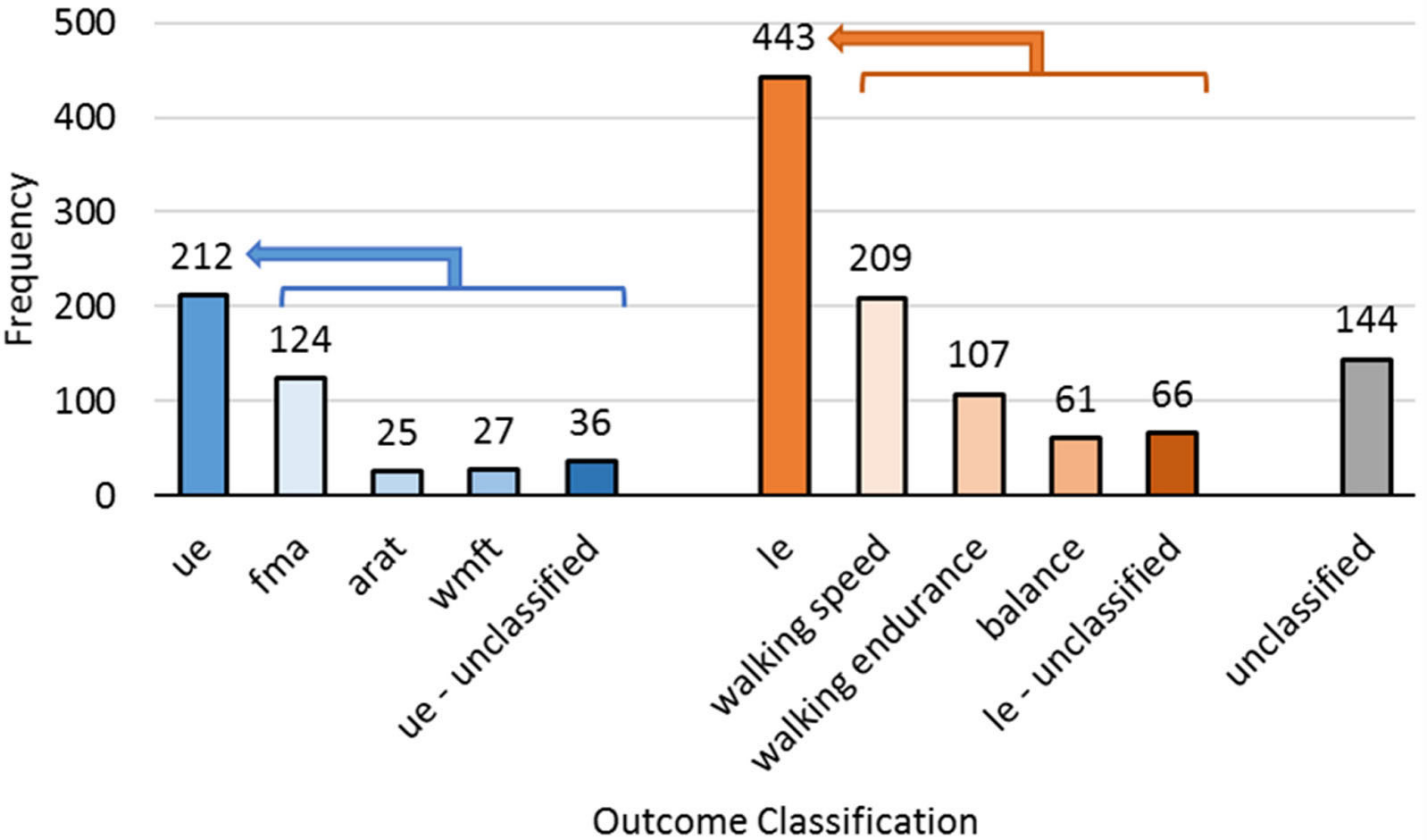
**The frequency of different outcome measures for the upper-extremity (ue, *n* = 212), lower-extremity (le, *n* = 443), and subscales of those two codes**. Complex whole body assessments that did not fit into one of these two categories were unclassified (*n* = 144).

There is a very wide range of outcome measures in SCOAR (*n* = 78 discrete outcome names) (3). Despite this multiplicity, all of the SCOAR outcomes are measures of impairment and/or motor function. Thus, these outcomes could be analyzed together in order to get a broad view of motor recovery outcomes. We have, however, built in several default filters in SCOAR to help researchers group common outcomes more quickly. The first filter is the “outcome_extremity” variable in the database, which codes outcomes based on the involvement of the upper-extremity (*ue*) and lower-extremity (*le*). Variables that do not cleanly fit into one of these categories (e.g., the Barthel Index or other activities of daily living scales) are left blank. Within the *ue* and *le* outcomes, we further subdivide outcomes with a second-level filter. Second-level filter codes for the upper-extremity in SCOAR include the Fugl–Meyer Assessment (*fma*), Action Research Arm Test (*arat*), and Wolf Motor Function Test (*wmft*). Second-level filter codes for the lower-extremity in SCOAR include measures of *walking speed* (including the 10-m walk test and variants thereof), *walking endurance* (including the 6 min walk test and variants thereof) and *balance* (which includes the Timed Up and Go Test and the Berg Balance Scale). The number of outcomes of each type is shown in Figure 3.

Naturally, some researchers may disagree with the filters/groupings, we have created for the defaults in the SCOAR database. As such, outcomes are also coded without a filter, using the name of the outcomes from the original study (the “outcome_name” variable in the database). In many cases, this unfiltered option will be appealing because it offers the user the greatest control over what information to pool together. However, if the user does not care which subscale of the FMA is being used (e.g., the ue total subscale, the wrist-hand subscale, or the shoulder-elbow subscale) then the second-level filter *fma* would be appropriate to use. Similarly, walking speed was measured in many different ways across the different studies. For example, walking speed was measured over 10, 8, or even 4.2 m, and at both self-selected velocity and maximal effort, but if the user is not concerned about such differences in these outcomes, then the second-level filter *speed* would be appropriate to use.

The wide variation in outcomes measures means that if a researcher wants to focus on a single outcome, it would greatly reduce the amount of data that can contribute to any single analysis. As with baseline CDEs, the reporting of so many different outcome measures with so few common outcome measures imposes severe limitations on hypothesis generation and data exploration. From the SCOAR database, there is a strong argument for consistent CDEs describing participant demographics, study methodology, and clinical outcomes. Panels of experts would be required to determine exactly what these CDEs should be (e.g., What are the best UE/LE outcome measures with respect to their sensitivity and clinical application?). While the exact battery of CDEs needs to be determined, it is clear that creating a comprehensive set of rehabilitation specific CDEs would enable researchers to ask innovative questions of existing data, bringing the results of hundreds of trials to bear on clinically relevant topics with unprecedented precision and statistical power.

### Data-Driven Searches and Novel Filters

A major advantage of organizing trial data in a central repository is that it can complement traditional bibliographic databases, such as PubMed or EMBASE. Traditional bibliographic tools assist authors in finding data from the top-down. That is, researchers arrive at data via well-designed searches using key terms, author names, medical subject headings, etc. By contrast, data-driven searches allow authors to find resources from the bottom-up. That is, SCOAR allows researchers to plot the relationships between variables and then click on large, outlying, or otherwise interesting data-points to obtain more information about that trial, such as patient demographics, type of intervention, and the reference for the published manuscript. For example, see http://tinyurl.com/SCOAR-datasearch.

In addition to these data driven searches, SCOAR enables researchers to constrain their searches by filters that are not available in bibliographic databases. For instance, a researcher could filter SCOAR to find only those trials that have used the ARAT as an outcome, in patients < 70 years old, and with sample sizes greater than 30. [Indeed, there are currently two studies in SCOAR that meet these specific criteria (51, 52).] Having these key variables describing participant demographics, research methodology, and clinical outcomes in an easily searchable database makes SCOAR a very useful compliment to existing bibliographic databases.

## Discussion

While SCOAR is a large step forward for data integration in stroke rehabilitation research, there are limitations associated with the database in its current form and limitations in using a database with “group-level” statistics more generally. One limitation is completeness; the data currently in SCOAR represent two waves of data extraction: the first wave extracted the first primary or secondary outcome measure that met our inclusion criteria while the second wave extracted any assessment based on the FMA or a measure of gait speed/endurance. This creates a representative, but not exhaustive extraction of all of the outcome measures used across the various studies. Currently, we are extracting the remaining outcomes from these RCTs, regardless of whether or not this outcome was primary or secondary. Once this third wave of data extraction is complete, SCOAR will contain all of the available summary statistics for any clinical measure of impairment or function used in these studies. We have also re-implemented our systematic search to update the database through 2016 and are in the process of extracting demographic, methodological, and statistical information from those studies.

Even as the evidence base in SCOAR expands, there is a major concern with the lack of CDEs in both outcome and baseline measures. From an information architecture perspective, reporting multiple outcomes per trial poses a unique problem. For instance, self-selected gait speed and maximal gait speed or various subscales of the FMA could all be reported in the same RCT. In SCOAR, this can be resolved by creating separate rows for each outcome, which is a relatively simple problem of *scale*. For the researcher, however, it is not always clear which measure is best or if one should average across multiple related measures, reflecting a more complicated problem of *ontology* (i.e., what are the fundamental constructs in rehabilitation and how do we measure them?) (53–55). This lack of CDEs is indicative of a larger problem for rehabilitation science as a field: researchers need to think critically about which measures to collect/analyze in order to reduce the risk of false positives (56–58) and (ideally) develop a set of common measures for key constructs (1, 2, 59) that would allow for greater data integration across trials.

Similarly, a major concern across trials was in the way that therapies were reported. For control therapies in particular, the descriptions of the control interventions were vague and under-reported in comparison to experimental interventions. Control therapies had approximately half of the words dedicated to their description as experimental therapies. This lack of detail might be acceptable if control therapies had sufficient references to support them (e.g., references to standard operating procedures or other published guidelines). However, this does not seem to be the case, as control interventions had about half of the number of references in their descriptions compared to experimental interventions, and less than one reference on average. (It should be noted that these word/reference counts were based only on the text in the Methods sections of the original papers.)

For both control and experimental therapies, there is also a concern about how the dose of therapy is reported across trials. At the moment, the only consistently reported measure was time scheduled for therapy and we stress that this measure gives only a rough indication of the amount of physical practice actually performed during therapy. It would be preferable to develop a system where the type, intensity, and volume of physical practice could be tracked for individual patients and consistently reported across studies. The development of such a system would be a large undertaking, but if there was a consistent mechanism/taxonomy for quantifying what exercises were done, at what intensity, and at what volume during therapy, this would help overcome problems with under-describing therapies. Such a taxonomy, if validated, would also improve our understanding of what the key “active-ingredients” actually are in physical and occupational therapy (60).

Another limitation of having a database of summary statistics, rather than individual patient data, is the “resolution” of the data available to researchers. Obviously this resolution would be finer if individual data were available for each patient in each trial (for example, see the Virtual International Stroke Trials Archive),^2^ but having a database of individual patient data raises privacy concerns that limit open-access to the database and (potentially) the type of variables that can be included in the database (i.e., data that may jeopardize anonymity). Thus, SCOAR is ultimately more “share-able” than individual patient data and potentially easier to maintain.

**Figure 4:**
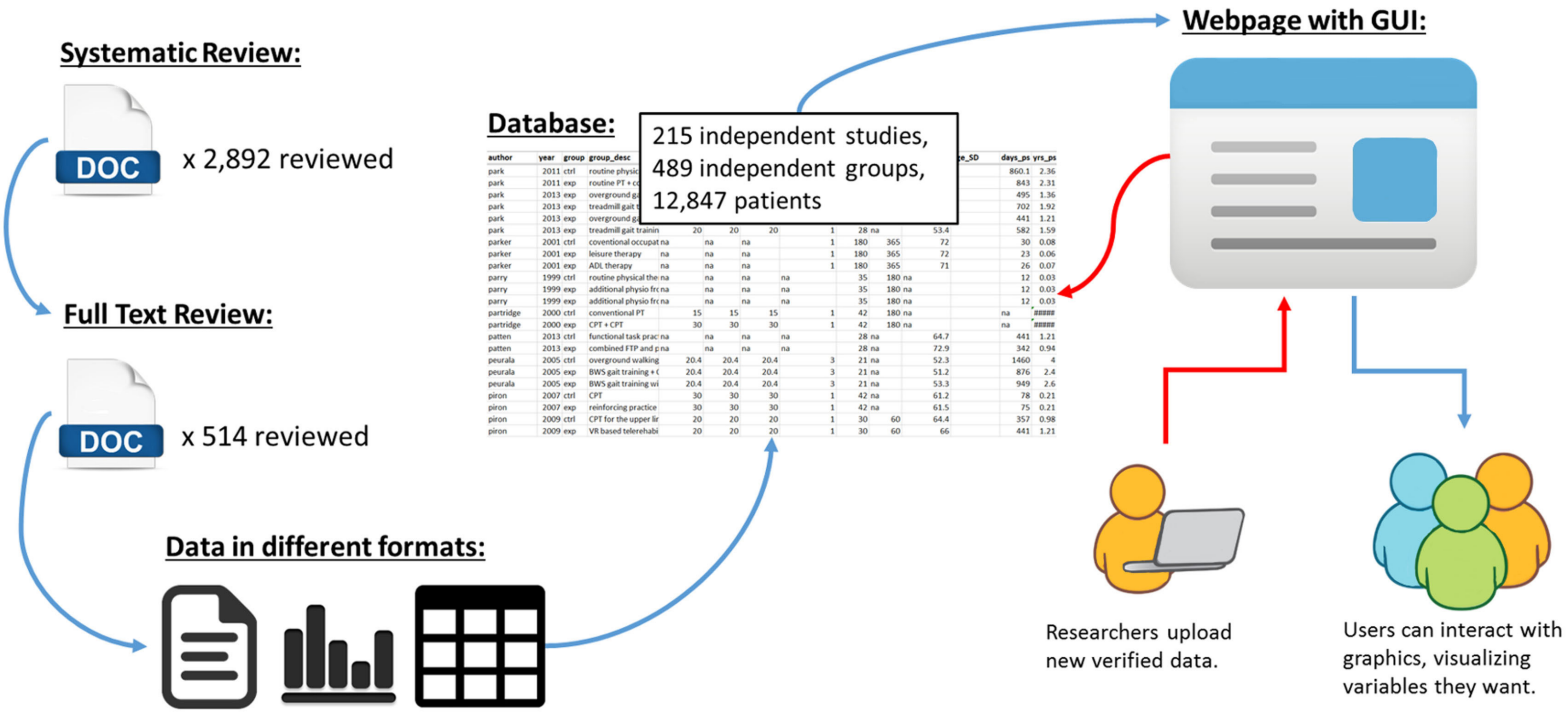
**Schematic showing the transformation of data from an unstructured format (i.e., separate texts, tables, and figures, often behind paywalls) to a structured format (i.e., SCOAR database) and our long-term goal of making SCOAR accessible to researchers through the internet**. This webpage would allow users to interact with the data (generating both statistical and graphical outputs) and allow researchers to upload new data (following a validation process) as new trials are published.

Finally, as shown in Figure 4, the long-term goal of SCOAR is to create a “living” database where researchers can add new trials to the database through the internet. We are currently working to develop a website with a graphical user interface that would exist on top of SCOAR, allowing researchers to visualize relationships between different variables in the database and/or download the raw data from SCOAR so that they can work with it offline. Furthermore, a major goal of this website is to allow researchers to upload new data as trials are published. By creating fillable forms that fit our data structure, we can make it very easy for researchers (e.g., the corresponding authors) to upload demographic information about their participants, methodological data about their intervention, and statistical information about their outcomes. This uploading would be validated by one of the SCOAR study personnel working with the author to ensure the quality/accuracy of the new data before it is officially added to SCOAR.

## Conclusion

The SCOAR database currently integrates demographic, methodological, and statistical data from 215 RCTs (representing 12,847 patients) that allows researchers to quickly visualize relationships between variables in motor rehabilitation for adults with stroke. Integrating data from 30+ years of published studies is certainly not trivial, but establishing this information architecture makes it easy to scale the database as new trials are published. In our own research, we are using SCOAR to analyze how the dose and timing of therapy interact to affect therapy outcomes, and by combing data from SCOAR with text-mining approaches we are exploring what “conventional” or “standard” therapy actually means in the context of RCTs (48). The open-access nature of SCOAR will help researchers and clinicians to (1) visualize relationships among variables based on the history of the field, (2) efficiently share data between trials, (3) generate hypotheses by allowing for exploratory meta-analyses, (4) search the current literature by complimenting existing bibliographic databases, and (5) design clinical trials by enabling more accurate and comprehensive power analyses.

## Author Contributions

KL, LB, and CL designed the systematic review and conceived the creation of the database. KL, SS, and AR oversaw the screening of manuscripts and data extraction. KL wrote the R code for management and analysis of the data. All authors contributed to the writing of the manuscript and approved the final version for publication.

## Conflict of Interest Statement

The authors declare that the research was conducted in the absence of any commercial or financial relationships that could be construed as a potential conflict of interest.

## Footnotes

^1^http://tinyurl.com/SCOAR-regions

^2^http://www.vista.gla.ac.uk/
