# Supplementary material for: Asking New Questions with Old Data: The Centralized Open-Access Rehabilitation Database for Stroke
Source: Front Neurol. 2016 Sep 20;7:153. doi: 10.3389/fneur.2016.00153 (PMC5028724; doi:10.3389/fneur.2016.00153)
Supplement: Supplementary file 1 [file data_sheet_1.docx]

**First Author: Year:**

**Title:**

**Journal:**

**Therapy:**

Group: Exp ______ (add a number if multiple experimental groups)

Ctrl

Brief description of therapy (i.e., ‘CIMT’, ‘body weight treadmill training’):

Page # on which therapy is described:

Hours/Day: Days/Week: Weeks: or Total Therapy:

How is time quantified: 1. Hours scheduled / 2. Time in therapy / 3. Active time / 4. Repetitions.

Time from Baseline to Terminal assessment: __________ days (“*Duration”*)

Time from Baseline to Follow Up assessment: __________ days (“*FollowUp”*)

**Participant demographics:**

Average age: Average Days Post Stroke:

Measures of initial severity (Measure) (Score)

1.

2.

3.

4.

Prior stroke (%, if available): Lesion Type (%, if available):

Exclusion criteria:

Page # on which criteria are described:

**“Primary” Outcome:**

Stated as primary outcome? **Y / N**

If secondary outcome was use, briefly state why (e.g., invalid stats):

Outcome Name: Units (if applicable): _______

Baseline Data:

n = _________ m = _________ s = _________

Terminal Assessment Data:

n = _________ m = _________ s = _________

Follow-up Assessment Data:

n = _________ m = _________ s = _________

*If change scores are given, list here:*

Δ(Baseline-Terminal):

mΔ = _________ sΔ = _________

Δ(Baseline-Follow-Up):

mΔ = _________ sΔ = _________

**Adverse Events and Drop-Outs:**

Terminal drop outs, n = _________ Follow-Up drop outs, n = ____________

Adverse Events reported (event type) (n)

1.

2.

3.

*Notes/Calculations:*
