# Supplementary material for: Asking New Questions with Old Data: The Centralized Open-Access Rehabilitation Database for Stroke
Source: Front Neurol. 2016 Sep 20;7:153. doi: 10.3389/fneur.2016.00153 (PMC5028724; doi:10.3389/fneur.2016.00153)
Supplement: Supplementary file 2 [file data_sheet_2.docx]

**Centralized Open-Access Rehabilitation database for Stroke (SCOAR).**

Data Dictionary

Updated 2016-03-25

Please direct questions regarding any variables in the dictionary to Keith Lohse ([rehabinformatics@gmail.com](mailto:rehabinformatics@gmail.com)).

**Variables in order of appearance:**

**“index”**

Numeric value indicating the absolute row on which a group of participants appears in the database.

**“study_id”**

Numeric value identifying separate randomized controlled trials in the database (note that ≥2 groups will come from the same study; at least one control and one experimental group).

**“id”**

Concatenated string combining the author and year handles.

**“author”**

The last name/surname of the first author. Entered all lowercase. In the case of follow-up studies being combined with an original publication the format will be “surname1/surname2”.

**“year”**

A four digit number indicating the year of publication. In the case of follow-up studies being combined with an original publication the format will be “year1/year2”.

**“group”**

A categorical variable indicating whether the data come from an experimental group, “exp”, or a control group “ctrl” as described in the original study.

**“group_id”**

Numeric value identifying an independent group of participants. Note that multiple groups of participants will come from the same study and that a single group might be measured on several outcomes in the database (i.e., there may be multiple outcomes per group).

**“group_desc”**

A short description of the treatment used in each group. These descriptions are entered by the data coder based on keywords used in the original publication. Common abbreviations/symbols include:

BWS = body-weight support

CIMT = constraint induced movement therapy

CPT = conventional physical therapy

FST = functional strength training

TT = treadmill training

VR = virtual reality

+ = one therapy supplemented with an additional, different therapy

**“url”**

A string variable providing the web address (usually the pubmed address) where researchers can access the original study.

**“country”**

A string variable indicating the country in which the randomized controlled trial was conducted, based on the contact information for the corresponding author.

**“region”**

A string variable indicating the region of the country in which the randomized controlled trial was conducted, based on the contact information for the corresponding author (i.e., states or provinces when applicable.

**“city”**

A string variable indicating the city in which the randomized controlled trial was conducted, based on the contact information for the corresponding author.

**“time_MAX”/ “time_50”/ “time_MIN”**

Given the issues with different types of therapy, it is not always clear how time was spent in therapy. In CIMT, for instance, participants might spend 5 hours per day under constraint for 5 days per week, for 4 weeks (100 total hours of constraint). To resolve this issue we have created three different calculations of total time:

time_MAX = a total time calculation where 100% of constraint time is counted as time in therapy.

time_50 = a total time calculation where 50% of constraint time is counted as time in therapy (we consider this calculation to be the most plausible as some, but not all, constraint time is counted).

time_MIN = a total time calculation where 0% of constraint time is counted as time in therapy.

**“detailed_time”**

A categorical variable describing the level of detail with which we know the time spent in therapy:

na = no time calculation feasible and/or available

1 = time scheduled for therapy is reported

2 = time that participants actually attended therapy is reported

3 = active time during therapy is reported (e.g., rest periods excluded)

4 = repetitions of therapy are reported

**“exp_dur”**

The duration of the intervention, in days, from the baseline assessment to the terminal assessment.

**“fu_dur”**

The duration, in days, from the baseline assessment to the latest follow-up assessment. Some studies might have multiple follow-ups, but we only extracted the longest follow-up duration.

**“age_base”**

Average age, in years, of the experimental/control group at the baseline assessment.

**“age_SD”**

[Sydney is interested in extracting this variable, we need to back track to do it.] The standard deviation of age, in years, of the experimental/control group at the baseline assessment.

**“days_ps”**

Patients’ chronicity. That is, the average time, in days, from the patient’s stroke to the beginning of the intervention.

**“days_cat”**

A categorical string variable indicating patients’ chronicity. This variable breaks days_ps into three groups <90 days, ≤365 days, and >365 days.

**“yrs_ps”**

The same as the days_ps variable, but in years instead of days.

**“stroke_type”**

A binary variable indicating if the proportion of ischemic strokes to hemorrhagic strokes is reported (1) or not reported (0).

**“nihss_base”**

Average National Institutes of Health Stroke Scale score at the baseline assessment.

**“mmse_base”**

Average Mini-Mental Status Exam score at the baseline assessment.

**“mMmse_base”**

Average modified Mini-Mental Status Exam score at the baseline assessment.

**“fim_base”**

Average Functional Independence Measure score at the baseline assessment.

**“brunstrom_base”**

Average Brunstrom Stages score at the baseline assessment.

**“frenchay_base”**

Average Frenchay Activities Assessment score at the baseline assessment.

**“fac_base”**

Average National Institutes of Health Stroke Scale score at the baseline assessment.

**“barthel_base”**

Average Barthel Index score at the baseline assessment.

**“mBarthel_base”**

Average modified Barthel Index score at the baseline assessment.

**“motricity_base”**

Average Motricity Index score at the baseline assessment.

**“BBS_base”**

Average Berg Balance Scale score at the baseline assessment.

**“BBT_base”**

Average Box and Block Test score at the baseline assessment.

**“gcs_base”**

Average Glasgow Coma Scale score at the baseline assessment.

**“MAS_base”**

Average Motor Activity Scale score at the baseline assessment.

**“sis_base”**

Average Stroke Impact Scale score at the baseline assessment.

**“rivermead_base”**

Average Rivermead Mobility Assessment score at the baseline assessment.

**“ashworth_base”**

Average Ashworth Spasticity Scale score at the baseline assessment.

**“mAshworth_base”**

Average modified Ashworth Spasticity Scale score at the baseline assessment.

**“rankin_base”**

Average Rankin Handicap Scale score at the baseline assessment.

**“bdi_base”**

Average Beck Depression Inventory at the baseline assessment.

**“cesd_base”**

Average Center Epidemiological Studies Depression Scale score at the baseline assessment.

**“ops_base”**

Average Orpington Prognostic Scale score at the baseline assessment.

**“terminal_dropout”**

The number of participants reported to have dropped out between the beginning and end of an intervention based on the description given in the text. If no estimate was possible, we inserted ‘na’.

**“outcome_name”**

The name of the outcome measure being recorded on that row. At the moment (2016-01-31) we have one outcome measure per study, but as the data base grows, we will have more. Common abbreviations include

6mwt: Six-Minute Walk Test

arat: Action Research Arm Test

bbs: Berg Balance Scale

fac: Functional Ambulation Category

fim: Functional Independence Measure

fma: Fugl-Meyer Assessment

fma-ue: Fugl-Meyer Assessment, upper extremity items only

fma-le: Fugl-Meyer Assessment, lower extremity items only

tug: Timed ‘Up and Go’ Test

wmft: Wolf Motor Function Test

**“outcome_extremity”**

This categorical string variable indicates if an outcome was a measure of upper extremity function (“ue”) or lower extremity function (“le”). In the event that an outcome could not be clearly categorized or was a composite of both lower and upper extremity function, then that cell was left blank.

**“ue_subscale”**

The categorical string variable breaks the measures of upper extremity function into their most common categories: the Action Research Arm Test (“arat”), the Fugl-Meyer Assessment (“fma”), and the Wolf Motor Function Test (“wmft”). Other cases are left blank.

**“le_subscale”**

The categorical string variable breaks the measures of lower extremity function into their most common categories. Variants of the 10 m walk test are coded as “speed”. Variants of the 6 min walk test are coded as “capacity”. The Timed-Up-and-Go Test and the Berg Balance Scale are coded as Balance.

**“SCOAR_outcome” & “p_outcome_stated”**

In constructing the SCOAR database, our goal was to take only validated clinical outcomes of function (e.g., no kinematics, physiologics, or esoteric variables unique to a particular study). We also wanted to select only the primary outcome for each study (i.e., the outcome measure the study was statistically powered to detect). However, many studies either did not list a primary outcome or some listed a kinematic measure as the primary outcome and a function measure was secondary. ***Our policy was always to take the first primary outcome that was a validated clinical measure and in the event no primary measure was available/state, the first eligible outcome was selected.***

Thus, the SCOAR outcome column denotes variables that were extracted in this first pass (‘lohse’) as the database grows, however, we hope to extract more outcomes and we will indicate by whom they were extracted. Similarly, ‘p_outcome_stated’ indicates if the measure was listed as primary outcome in the original text (1) or not (0).

**“itt_analysis”**

A value of 1 is given only if the authors explicitly state that their analysis followed an “intention to treat” principle or explicitly stated that they handled missing values by carrying the last available observation forward.

**“base_n”**

Given the description in the text, this is the number of participants whose data contribute to the baseline mean (base_m) and baseline standard deviation (base_sd) calculations. Note that this is not necessarily the number of participants randomized to each group, depending on how the authors conducted their analysis.

**“base_m”**

Mean of the outcome measure at baseline.

**“base_sd”**

Standard deviation of the outcome measure at baseline.

**“difference_score”**

Indicates whether or not terminal outcomes were reported as terminal/follow-up means or as change from baseline.

**“term_n”**

Given the description in the text, this is the number of participants whose data contribute to the terminal mean (term_m) and terminal standard deviation (term_sd) calculations. Note that this is not necessarily the number of participants who completed the intervention, depending on how the authors conducted their analysis.

**“term_m”**

Mean of the outcome measure at the terminal assessment (i.e., end of intervention).

**“term_sd”**

Standard deviation of the outcome measure at the terminal assessment (i.e., end of intervention).

**“diff_base”**

Is the follow-up outcome being listed as a difference from baseline? 1 = yes; 0 = no.

**“diff_term”**

Is the follow-up outcome being listed as a difference from the terminal assessment? 1 = yes; 0 = no.

**“fu_n”**

Given the description in the text, this is the number of participants whose data contribute to the follow-up mean (fu_m) and follow-up standard deviation (fu_sd) calculations. Note that this is not necessarily the number of participants who completed the follow-up assessment, depending on how the authors conducted their analysis.

**“fu_m”**

Mean of the outcome measure at the latest long-term follow-up.

**“fu_sd”**

Standard deviation of the outcome measure at the latest long-term follow-up.

**“est_corr”**

The estimated correlation between time points. In general, this correlation is not reported in clinical trials of rehabilitation. However, when raw data were presented or authors reported the standard deviation at pre-test, the standard deviation at post-test, and the standard deviation of the difference, the correlation coefficient could be calculated. Based on these data, the pre- to post-test correlation is generally large and positive (>0.6), as such, we make the default estimate more conservative (r = 0.5), which increases or variance calculations (term_Vd and fu_Vd below), ultimately widening the confidence intervals in our models.

**“term_diff”**

The difference between the mean terminal assessment and the mean baseline assessment. Used in the calculation of the terminal Cohen’s d.

**“term_s_diff”**

The pooled standard deviation (combing the SD from the pre- and post-test). Used in the calculation of the terminal Cohen’s d.

**“fu_diff”**

The difference between the mean follow-up assessment and the mean baseline assessment. Used in the calculation of the follow-up Cohen’s d.

**“fu_s_diff”**

The pooled standard deviation (combing the SD from the pre- and follow-up test). Used in the calculation of the follow-up Cohen’s d.

**“term_d”**

Cohen’s d for the change from the baseline assessment to the terminal assessment (term_diff/term_s_diff). Subtraction was arranged so that positive values always reflect improvement from baseline.

**“fu_d”**

Cohen’s d for the change from the baseline assessment to the follow-up assessment (fu_diff/fu_s_diff). Subtraction was arranged so that positive values always reflect improvement from baseline.

**“j_corr_factor”**

Given the number of large effect sizes and small effect sizes found in the which reasoned it would be best to convert Cohen’s d into Hedges’ g, which corrects for the fact that Cohen’s d tends to over-estimate effect sizes in small samples (see Borenstein et al., 2009)

$$g=J\times d$$

Where:

$$J= 1-\frac{3}{4\left( n-1 \right)-1}$$

**“term_g”**

The terminal Hedges’ g (i.e., terminal Cohen’s d multiplied by the correction factor). Subtraction was arranged so that positive values always reflect improvement from baseline.

**“fu_g”**

The follow-up Hedges’ g (i.e., follow-up Cohen’s d multiplied by the correction factor). Subtraction was arranged so that positive values always reflect improvement from baseline.

**“term_Vd”**

Variance of the terminal Cohen’s d (see Borenstein et al., 2009). Where n is the number of participants at baseline and rest is the estimated correlation between pre-test and post-test. (Correlations were estimated based on other sources if no exact correlation was calculable for that group.)

$$V_{d}= \left( \frac{1}{n}+\frac{d^{2}}{2n} \right)2\left( 1-r_{est} \right)$$

**“fu_Vd”**

Variance of the terminal Cohen’s d (see Borenstein et al., 2009).

**“term_Vg”**

Variance of the terminal Hedges’ g (see Borenstein et al., 2009).

$$V_{g}=V_{d}\times J^{2}$$

**“fu_Vg”**

Variance of the follow-up Hedges’ g (see Borenstein et al., 2009).

**“chck2”**

Indicates that ***all of the data*** for that group have been confirmed by at least two independent coders.

**“notes”**

Any additional information that the extraction team felt was relevant to that group. (Note this is not an exhaustive list of all of the notes/calculations from the hard-copy data extraction forms. Only the most relevant notes/calculations were included in the electronic database.)
